# Field safety and efficacy study with a cannabidiol/cannabidiol acid-rich hemp paste in cats with osteoarthritic pain

**DOI:** 10.1177/1098612X251367629

**Published:** 2025-10-15

**Authors:** Liza M Mulder, Jeanine Deterd Oude Weme, Niels R Blees, Joseph J Wakshlag, Daniel Hughes, Ronald Jan Corbee

**Affiliations:** 1 Department of Clinical Sciences, Faculty of Veterinary Medicine, Utrecht University, Utrecht, The Netherlands; 2 Anicura de Tweede Lijn, Zwolle, The Netherlands; 3 ElleVet Sciences, South Portland ME, The Netherlands

**Keywords:** Pain management, cannabidiol, CBD pharmacokinetics, surgery, osteoarthritis, nutritional supplements, orthopaedics

## Abstract

**Objectives:**

Feline osteoarthritis (OA) is a common, degenerative joint condition that is an important cause of chronic pain in cats. Cannabinoids have shown potential in reducing pain and inflammation in OA, but research in cats is limited. This study aimed to assess the safety and efficacy of a cannabidiol (CBD)/cannabidiol acid (CBDA) paste would in cats with OA. We hypothesised that CBD/CBDA paste would reduce pain scores and would be safe for use without significant adverse effects.

**Methods:**

In this randomised, double-blinded, placebo-controlled crossover study, client-owned cats with OA were assigned to two study groups and received a 6-week course of both CBD/CBDA paste and a placebo. During the initial consultation and following appointments, cats underwent orthopaedic examination pain assessment with the North Carolina State University Translational Research in Pain (TRiP) Feline Musculoskeletal Pain Index scoring system and blood examination. Owners filled in a bi-weekly questionnaire (Dutch Orthopaedic Rating for Feline Osteoarthritis Pain, DORFOP). TRiP and DORFOP scores were analysed using mixed-effects models. Clinical chemistry in week 6 was compared using paired *t*-tests.

**Results:**

A total of 26 cats participated in the study. Of these, 12 dropped out primarily because of their refusal to eat the CBD/CBDA paste, and, in some cases, vomiting was reported. Fourteen cats were left to complete the study. CBD/CBDA paste significantly reduced TRiP and DORFOP scores after 6 weeks of administration, with changes in DORFOP starting after 2 weeks. No differences in clinical biochemistry were observed between the placebo and CBD/CBDA paste at week 6.

**Conclusions and relevance:**

CBA/CBDA paste effectively reduced pain in cats, but the high drop-out rate is concerning. Further research with larger sample sizes and longer treatment durations is needed to confirm these findings.

## Introduction

Osteoarthritis (OA) is a degenerative disease affecting the synovial joints, with significant implications for the health and wellbeing of cats. OA affects 16.5–91% of all cats and can be associated with chronic pain, impaired mobility and a diminished quality of life.^1–4^ Despite this high prevalence, clinical signs may be subtle and mobility impairments often remain undiagnosed in cats, complicating the clinical identification of this condition.^5–7^ Treatment modalities for OA often combine the use of medication, such as non-steroidal anti-inflammatory drugs, weight loss and dietary supplements.^4–6^ However, the long-term use of these therapies is often limited by potential side effects or low compliance, highlighting the need for safer and more sustainable alternatives.^7^

Recently, there has been growing interest in cannabidiol (CBD)-based therapies for their multimodal analgesic and anti-inflammatory properties.^8–10^ Cannabis-derived compounds have a long history of medicinal use; however, its psychotropic effects, attributed to delta-9-tetrahydrocannabinol (THC), led to the criminalisation of cannabis in the 20th century in some countries. In recent decades, the legalisation of hemp, rich in CBD but with low THC levels, has reignited interest in its therapeutic potential.^11^ CBD and its native form found in plants were shown to be effective in reducing signs of pain perception in dogs and steers.^12,13^ CBD, and its precursor cannabidiol acid (CBDA), are the primary non-psychoactive cannabinoids found in hemp. Both CBD and CBDA interact with the endocannabinoid system to modulate pain and inflammation.^14^ Their mechanisms of action involve various molecular targets, including transient receptor potential channels, serotonin receptors, orphan G-protein receptors and peroxisome proliferator-activated receptors.^15^

The pharmacokinetics and safety of CBD-rich oil in cats have been previously investigated. Short-term studies (4 weeks) reported elevated alanine transaminase (ALT) levels in 3/9 cats, accompanied by lethargy and/or inappetence, but no elevations were observed in 10 cats during a 26-week study using a dosage of 4 mg/kg q24h.^16^ Another study found no significant changes in complete blood count or biochemistry with CBD administration of up to 30.5 mg/kg CBD oil and up to 41.5 mg/kg THC oil for 33 days in 16 cats.^17^ A trial using a CBD/CBDA extract (2 mg/kg q12h for 3 months) reported no population-wide liver enzyme elevations, although 1/8 cats had elevated ALT.^18^ A 2023 case report on CBD for osteoarthritic pain (0.5–0.25 mg/kg) noted sedative effects and ALT elevation, likely influenced by the product’s high THC content.^10^

Previous studies on CBD’s effects in dogs with OA have shown promising results, with significant improvements in quality of life, as assessed by owners and veterinarians.^19^ However, to the best of the authors’ knowledge, placebo-controlled clinical studies on the use of CBD as an analgesic for OA in cats remain limited.^10,15^ Therefore, this study aimed to investigate the safety and efficacy of a pharmacokinetically tested CBD/CBDA-rich hemp paste in cats with naturally occurring OA through a randomised, double-blind, placebo-controlled, crossover study. We hypothesised that treatment with CBD/CBDA-rich hemp paste would result in significant reductions in pain scores in cats with OA. In addition, we hypothesised that CBD/CBDA-rich hemp paste would be safe for use in cats and would not cause significant changes in blood parameters.

## Materials and methods

### Animals

Adult cats (age >1 year) with a medical history and clinical signs suggesting OA were prospectively recruited via social media and direct contact with veterinary students, university staff, referring veterinarians, breeders and catteries. OA was confirmed by orthopaedic examination and radiographic imaging. Inclusion criteria were the combination of radiographic evidence of OA in one or more synovial and/or non-synovial joints, clinical signs of pain and mobility impairments attributable to OA. Cats with concurrent systemic and neurological comorbidities were excluded, based on clinical examination and blood analysis. All participants were otherwise healthy based on veterinary examination and bloodwork, including sodium, potassium, chlorine, blood urea nitrogen (BUN), creatinine, calcium, phosphorus, magnesium, glucose, aspartate transaminase (AST), ALT, alkaline phosphatase (ALP), gamma-glutamyl transferase (GGT), bilirubin, total protein, albumin, globulin, cholesterol and creatine kinase (CK). Priorly instituted OA management was continued, provided no changes in treatment or diet occurred during the trial or 2 weeks prior. Owners signed informed consent forms. The study was approved by the National Animal Care Committee (registration number AVD10800202216205WP3-02).

### Treatment protocol

The CBD/CBDA paste (ElleVet Sciences) was formulated specifically for feline use, with a cannabinoid concentration of approximately 5 mg/g of CBD and 5 mg/g of CBDA (post-trial analysis revealed 10.8 mg/g total cannabinoids). The paste was validated for cannabinoid content, and all products were confirmed free from solvents, pesticides, microbes, heavy metals and mycotoxins by a third-party laboratory. The dosage was aimed at 4 mg CBD + CBDA per kg body weight daily, divided into two doses (1 g of paste per dose or 2 g of paste per dose depending on the cat’s weight), and owners were instructed on using Dial-a-Dose syringes to dose the paste and administer it orally. The placebo paste matched the CBD/CBDA paste in appearance and consistency to maintain blinding. In addition, 0.1% myrcene, a major terpene, was added to the placebo to match the odour profile of the CBD/CBDA-containing paste.

### Study design

The study was designed as a multicentre randomised, double-blinded, placebo-controlled, crossover study that took place across multiple referral clinics across the Netherlands. Cats were randomly assigned to two groups. Group 1 received a CBD/CBDA paste for 6 weeks, followed by a 2-week washout and 6 weeks of the placebo, so each cat served as their own control. Group 2 received treatments in the reverse order, starting with the placebo.

Veterinary consultations were conducted at four time points: baseline (week 0) and follow-ups at weeks 6, 8 and 14. Initial assessments included detailed medical history, orthopaedic examination, radiographic imaging to confirm OA and blood tests. The blood panel included sodium, potassium, chlorine, BUN, creatinine, calcium, phosphorus, magnesium, glucose, AST, ALT, ALP, GGT, bilirubin, total protein, albumin, globulin, cholesterol and CK. Follow-up consultations repeated orthopaedic examinations and blood analyses to monitor safety and adverse reactions. Serum samples were collected at each time point for cannabinoid analysis to verify systemic delivery of CBD/CBDA. Cats were monitored closely for adverse effects (clinical signs and laboratory data), and the severity of any noted adverse effects was classified subjectively.

### Pain scoring and owner assessments

Orthopaedic pain was quantified during veterinary examinations using the North Carolina State University Translational Research in Pain (TRiP) Feline Musculoskeletal Pain Index scoring system (see file 1 in the supplementary material). Each joint was palpated for pain and assessed for crepitus, effusion and thickening.^20,21^

Owners completed the Dutch Orthopaedic Rating for Feline Osteoarthritis Pain (DORFOP) scale bi-weekly throughout the study (see file 2 in the supplementary material). The DORFOP, a unidimensional scale, with a range of 6–30, evaluates mobility and behavioural indicators of OA pain. Higher scores indicate more severe impairment. In addition, a custom questionnaire (see file 2 in the supplementary material) was used to evaluate changes in each cat’s condition relative to its baseline at the start of the study, with responses ranging from ‘much better’ to ‘much worse’ as the five Likert-scale answers.^22^

### Serum cannabinoid analysis

Serum cannabinoids and their metabolites, including CBD, CBDA, delta-9 (D9)-THC and others, were quantified using a liquid chromatography/tandem mass spectrometry (LC/MS/MS) method (ElleVet Sciences). Serum preparation involved adding 100 µl serum to acetonitrile and internal standards, followed by centrifugation. Samples were analysed on an Agilent LC-MS/MS system with a Poroshell 120 SB-C18 column. Calibration curves for each cannabinoid were generated using matrix-matched standards, and concentrations were calculated using linear regression with 1/c weighting, as in the study by Wang et al.^23^ The lower limit of detection and quantitation in cat serum is provided in Table 1.

**Table 1:** Lower limits of detection and quantitation for cannabinoids in cat serum (liquid chromatography/tandem mass spectrometry method)

Compound name	Precursor ion	Production	Polarity	LOD (ng/ml)	LOQ (ng/ml)
11-NOR-COOH-D9-THC-GLUCURONIDE	519.56	343.1, 299.0	Negative	2.5	5
11-OH-D9-THC	331.47	193.3, 313.8, 122.9	Positive	0.5	1.0
6-OH-CBD	329.45	173.1, 311.1, 157.8	Negative	0.5	1.0
7-COOH-CBD	343.43	297.0, 299.1, 231.0	Negative	0.5	1.0
7-OH-CBD	329.45	261.1, 311.1, 178.9	Negative	0.5	1.0
CBC	315.47	193.4, 123.1, 93.1	Positive	2.5	5
CBCA	357.46	339.1, 313.1, 191.0	Negative	0.5	1.0
CBD	315.47	193.4, 123.3, 93.0	Positive	0.5	1.0
CBDA	359.48	219.4, 341.3, 261.5	Positive	5	5
CBGA	361.5	219.4, 149.1, 135.1	Positive	1	2.5
D9-THC	315.47	193.4, 123.1, 93.0	Positive	2.5	5
THCA	357.46	313.1, 245.0, 191.0	Negative	0.5	1.0
D3-11-OH-D9-THC	334.49	316.4, 196.3, 105.3	Positive	N/A – internal standards	
D3-7-OH-CBD	332.7	314.2, 172.9, 264.1	Negative	N/A – internal standards	
D3-CBD	318.49	196.3, 123.2, 93.1	Positive	N/A – internal standards	
D3-D9-THC	318.49	196.4, 123.2, 93.0	Positive	N/A – internal standards	
D3-THCA	360.49	316.1, 248.0, 194.0	Negative	N/A – internal standards	
D3-7-COOH-CBD	346.45	302.2, 300.1, 234.1	Negative	N/A – internal standards	
D3-CBC	318.49	196.5, 123.1, 81.0	Positive	N/A – internal standards	
D3-CBCA	360.49	316.1, 248.0, 194.0	Negative	N/A – internal standards	
D3-CBG	320.51	196.4, 123.3, 95.2	Positive	N/A – internal standards	
D3-CBGA	364.52	346.8, 222.4, 77.0	Positive	N/A – internal standards	
D3-CBDA	362.5	344.8, 222.5, 91.1	Positive	N/A – internal standards	

11-NOR-COOH-D9-THC-GLUCURONIDE = 11-nor-9-carboxy-D9-tetrahydrocannabinol glucuronide; 11-OH-D9-THC = 11-hydroxy-D9-tetrahydrocannabinol; 6-OH-CBD = 6-hydroxycannabidiol; 7-COOH-CBD = 7-carboxycannabidiol; 7-OH-CBD = 7-hydroxycannabidiol; CBC = cannabichromene; CBCA = cannabichromenic acid; CBD = cannabidiol; CBDA = cannabidiolic acid; CBGA = cannabigerolic acid; D9-THC = D9-tetrahydrocannabinol; THCA = tetrahydrocannabinolic acid; D3-11-OH-D9-THC = deuterated 11-hydroxy-D9-tetrahydrocannabinol; D3-CBD = deuterated cannabidiol; D3-11-OH-D9-THC = deuterated 11-hydroxy-D9-tetrahydrocannabinol; D3-7-OH-CBD = deuterated 7-hydroxycannabidiol; D3-D9-THC = deuterated D9-tetrahydrocannabinol; D3-7-COOH-CBD = deuterated 7-carboxycannabidiol; D3-CBC = deuterated cannabichromene; D3-CBCA = deuterated cannabichromenic acid; D3-CBG = deuterated cannabigerol; D3-CBGA = deuterated cannabigerolic acid; D3-CBDA = deuterated cannabidiolic acid; LOD = limit of detection; LOQ = limit of quantification; N/A = not available

### Statistical analysis

All continuous variables were summarised using descriptive statistics. Data were checked for normality using Q–Q plots and Shapiro–Wilk tests. Data were reported as median (range). Clinical chemistry and serum cannabinoids findings are reported as mean ± SD. Statistical analyses were performed using R version 4.4.1 (R Core Team, 2023). Clinical chemistry for both treatments at week 6 were compared using paired *t*-tests. The influence of treatment on DORFOP scores and TRiP scores, both total scores and individual domains, was evaluated using random intercepts models. A step-up selection model was used to fit fixed factors treatment, week, use of concurrent treatment, age, breed, sex, treatment order and interactions, with the individual animal as random effect. Baseline data were included as covariate to correct for baseline levels.^24^ Akaike’s information criteria and likelihood-ratio tests were used to compare models. Pairwise post-hoc comparisons were corrected by false discovery rate. The assumptions for mixed-effects models (ie, normality of residuals, homogeneity of variance and independence of random effects) were met for each model. Only the data of cats that concluded the clinical study were included in analyses. Significance was set at *P* <0.05.

## Results

### Animals

**Figure 1:**
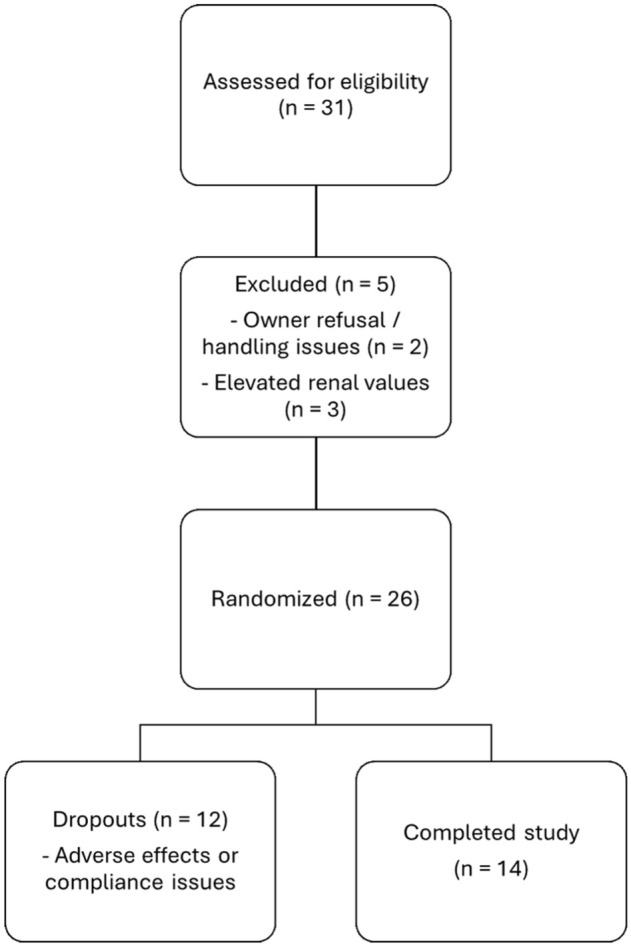
CONSORT diagram of the study population. Flow of cats through the study, including screening, randomisation, dropouts and study completion

A total of 26 cats were enrolled in the study. Detailed characteristics of the included animals, treatment group assignment and reason for dropout are summarised in Tables 2 and 3. Four animals used concurrent analgesia during the study, and these animals were evenly distributed over each treatment groups. OA was predominantly present in the hips, elbows, lumbar and lumbosacral vertebrae, and stifles. Before the start of the study, 31 cats were assessed for eligibility. Five cats were excluded: two because of owner refusal of blood sampling or poor handling tolerance, and three owing to elevated renal values detected in their medical records. A total of 26 cats were subsequently enrolled and randomised into treatment groups. Ultimately, 14 cats successfully completed the study. The detailed flow of participants is depicted in the CONSORT diagram (Figure 1).

**Table 2:** Cats enrolled in the study, including their sex, neutering status, age, breed distribution and initial treatment group assignment

Category	Description	Value
Sex and neutering status	Female spayed	6 (23)
	Male castrated	16 (61.5)
	Female intact	1 (4)
	Male intact	3 (11.5)
Age	Median	10 years 4 months
	Range	2 years 1 month to 17 years 7 months
Breeds	Domestic shorthair	10 (38)
	British Shorthair	6 (22)
	Maine Coon	5 (20)
	Scottish Fold	2 (8)
	British Longhair	1 (4)
	Norwegian Forest Cat	1 (4)
	Sphynx	1 (4)
Treatment group enrolment	Group 1	14 (54)
	Group 2	12 (46)

Data are n (%) unless otherwise indicated

**Table 3:** Overview of cats participating in the study, including their characteristics, treatment group assignments and reasons for dropout (if applicable)

Cat	Sex	Breed	Age (in years)	Weight (kg)	Neutering status	Group	Dosage* (mg/kg/day)	Dropout	Reason for dropout	Treatment at the time of dropout
1	Female	DSH	17	5.3	Spayed	2	3.77	No	–	–
2	Male	BSH	17	5.5	Castrated	1	3.64	No	–	–
3	Female	DSH	2	3.4	Spayed	1	5.89	Yes	Lethargy	CBD/CBDA
4	Male	Sphynx	8	4	Castrated	2	5	No	–	–
5	Male	BSH	3	5.3	Castrated	2	3.77	No	–	–
6	Male	BLH	9	4.7	Castrated	1	4.26	Yes	Vomiting	CBD/CBDA
7	Male	DSH	15	6	Castrated	2	3.33	Yes	Vomiting	CBD/CBDA
8	Male	Maine Coon	5	8.4	Intact	1	2.38	No	–	–
9	Male	Maine Coon	9	10.2	Castrated	1	3.92	Yes	Did not accept the paste	CBD/CBDA
10	Female	Maine Coon	7	8.3	Spayed	1	2.4	No	–	–
11	Male	DSH	5	5.8	Castrated	1	3.45	No	–	–
12	Male	DSH	10	4.4	Castrated	2	4.55	Yes	Vomiting	CBD/CBDA
13	Male	DSH	14	5	Castrated	2	4	Yes	Owner related	Placebo
14	Male	Maine Coon	11	6.2	Castrated	1	3.23	Yes	Vomiting	CBD/CBDA
15	Male	Maine Coon	3	6.4	Intact	1	3.13	No	–	–
16	Male	BSH	9	10.5	Castrated	2	3.8	Yes	Inappetence	CBD/CBDA
17	Female	Scottish Fold	5	4.3	Spayed	2	4.65	Yes	Vomiting and diarrhea	CBD/CBDA
18	Male	DSH	11	4.2	Castrated	2	4.76	No	–	–
19	Female	DSH	12	3.8	Spayed	2	5.26	No	–	–
20	Male	BSH	9	5.2	Castrated	1	3.85	No	–	–
21	Male	BSH	10	4.8	Castrated	1	4.17	No	–	–
22	Female	DSH	13	4.5	Spayed	2	4.44	No	–	–
23	Male	BSH	16	6.6	Castrated	2	3.03	Yes	Vomiting	Washout period
24	Male	DSH	11	5.3	Castrated	1	3.77	Yes	Did not accept the paste	Placebo
25	Female	DSH	17	5.8	Intact	1	3.45	Yes	Vomiting	CBD/CBDA
26	Male	Norwegian Forest Cat	13	7.66	Castrated	1	3.06	No	–	–

* Dosages have been doubled for cats weighing >8.5 kg

BSH = British Shorthair; CBD = cannabidiol; CBDA = cannabidiol acid; DSH = domestic shorthair

### Randomisation and blinding

Cats were alternately assigned to treatment sequences (CBD/CBDA first or placebo first) based on the order of enrolment ensuring balanced group allocation. The study was conducted in a blinded manner: both the investigators and the cat owners were unaware of the treatment content during the administration period.

### Dropout

A total of 12 cats dropped out of the study. All dropouts except for two were receiving the paste containing CBD/CBDA (Table 3). The dropout that received the placebo did not complete the study because of owner-related issues. The most common reason for dropout was gastrointestinal issues; 7/12 (58%) cats discontinued participation because of vomiting and/or diarrhoea. Two (16%) cats refused to consume any of the pastes, which led to dropout. Another cat (8%) stopped eating, resulting in anorexia and subsequent dropout. In addition, 2/12 (16%) cats experienced behavioural changes while on medication, which led to their discontinuation from the study.

### DORFOP scores

**Figure 2:**
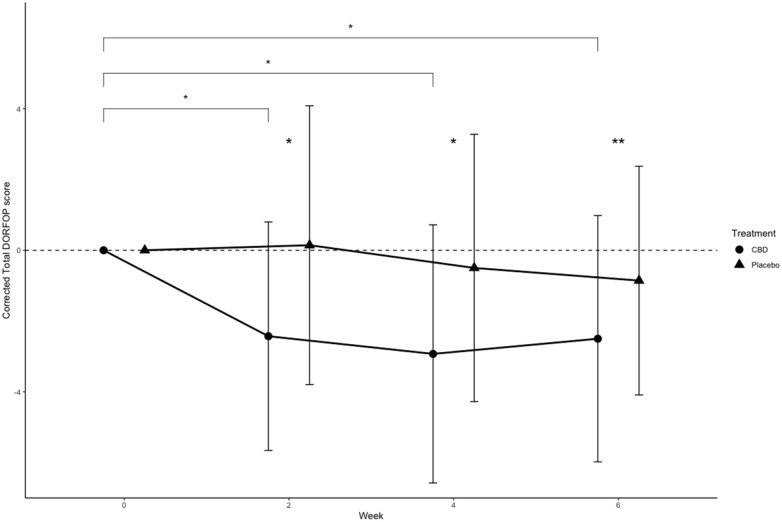
Total Dutch Orthopaedic Rating for Feline Osteoarthritis Pain (DORFOP) scores corrected at each measurement by baseline values at week 0 (n = 14). Dots and error bars represent mean ± SD. Brackets with significant markers indicate between-time point difference for a treatment. Significant markers at time point indicate between-treatment difference. *P <0.05 between treatments, **P <0.01. CBD = cannabidiol

The owner assessment through DORFOP scores changed significantly compared with baseline week 0 during CBD/CBDA treatment at weeks 2, 4 and 6, but not for the placebo (Figure 2). This resulted in significantly lower total DORFOP scores during CBD/CBDA treatment compared with the placebo from week 2 and onwards, but not at baseline. In addition, none of the other included fixed factors influenced the model.

**Figure 3:**
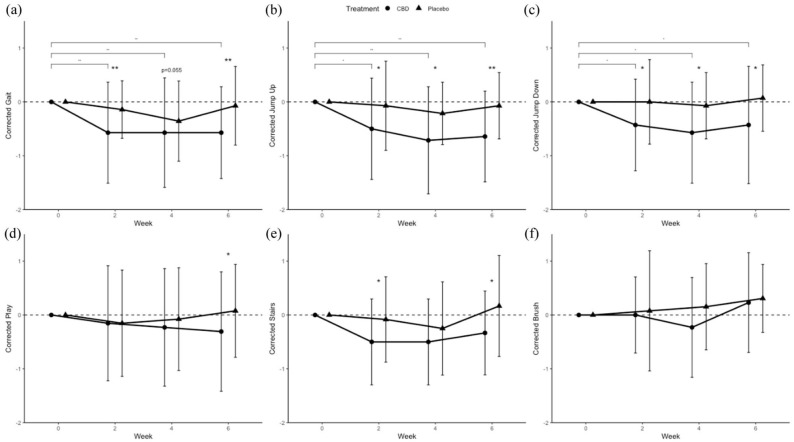
Domain-specific Dutch Orthopaedic Rating for Feline Osteoarthritis Pain (DORFOP) scores for (a) gait (n = 14), (b,c) jumping up and off objects (n = 14), (d) playing (n = 13), (e) walking upstairs (n = 8) and (f) brushing (n = 8), with each measurement corrected by baseline values at week 0. Dots and error bars represent mean ± SD. Brackets with significant markers indicate between-time point difference for a treatment. Significant markers at time point indicate between-treatment difference. *P <0.05, **P <0.01. CBD = cannabidiol

All individual DORFOP domains, except for brushing, showed similar effects of treatment (Figure 3). DORFOP scores for gait and walking on stairs during CBD/CBDA treatment were reduced compared with placebo at weeks 2 and 6, but not at week 4. DORFOP scores for jumping up and down were significantly lower than placebo at weeks 2, 4 and 6. DORFOP play scores were lower for CBD/CBDA than placebo in week 6, but not in the others. None of the DORFOP domains had differences between treatments at baseline, and no other factors aside from ‘Treatment’ and ‘Week’ improved the models for each domain.

Incomplete responses were observed in certain DORFOP domains, particularly those related to brushing and stair climbing, in 6/14 cats that completed the study and in 3/12 cats that did not. This was primarily due to environmental factors, such as the absence of stairs in the home or the cat not being accustomed to brushing.

### TRiP scores

**Figure 4:**
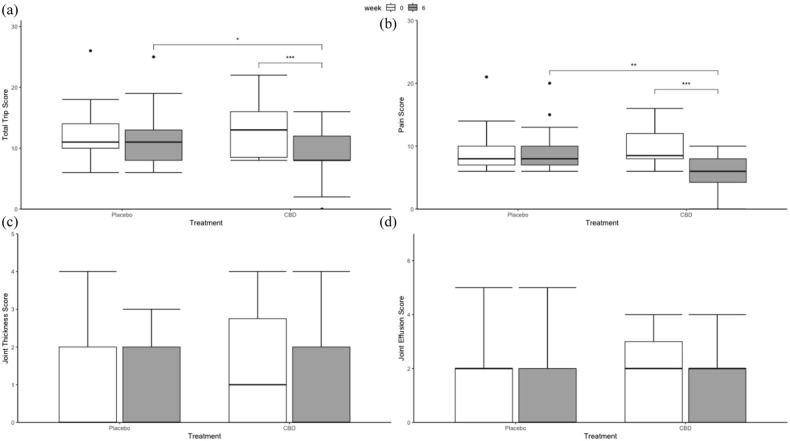
Box plots of (a) total Translational Research in Pain (TRiP) score and (b–d) domain-specific TRiP scores at week 0 and week 6 for placebo and cannabidiol (CBD)/cannabidiol acid (CBDA) paste (n = 14). The box represents the median and 25th and 75th quantile, Whiskers extend from the hinge to the smallest and largest value at most 1.5 × interquartile range of the hinge. *P <0.05, **P <0.01, ***P <0.001

Total TRiP scores were only affected by the interaction between ‘Treatment’ and ‘Week’ (Figure 4). Pairwise comparisons revealed that utilisation of the CBD/CBDA paste reduced TRiP scores compared with baseline, resulting in significantly lower scores compared with placebo (*P* <0.05) at week 6, but not at baseline. Similarly, domain scores for TRiP pain were significantly lower in CBD/CBDA-treated animals compared with placebo at week 6. No other factors, such as age and breed, influenced these models. Yet, TRiP effusion, joint thickening and crepitus scores were not affected by CBD/CBDA treatment.

### Clinical chemistry

None of the animals displayed significant treatment-related changes in clinical chemistry parameters at week 6 compared with baseline (Table 4). Incidental abnormalities were found compared with the reference intervals, including elevated chloride, glucose, total protein, albumin and gamma-globulins in all cats, regardless of treatment. These changes were not associated with clinical signs and are likely attributable to biological variation or stress-related responses, such as mild elevations in glucose, and were therefore considered incidental abnormalities without clinical relevance. For example, one cat had a chloride concentration of 115 mmol/l (reference interval [RI] 105–112) and another had a glucose level of 6.4 mmol/l (RI 3.4–5.7).

**Table 4:** Serum chemistry of cats receiving placebo or cannabidiol (CBD)/cannabidiol acid (CBDA) paste

		Placebo				CBD/CBDA paste			
		Week 0		Week 6		Week 0		Week 6	
		N	Mean ± SD	n	Mean ± SD	n	Mean ± SD	n	Mean ± SD
Liver	ALT	12	51.42 ± 11.1	11	53.55 ± 19.2	12	53.58 ± 19.4	12	52.08 ± 14.6
	AST	14	23.71 ± 6.1	13	23.46 ± 5.1	14	28.29 ± 9.0	14	25.07 ± 7.9
	ALP	13	33.08 ± 13.6	13	31.62 ± 13.5	13	30.08 ± 17.6	14	34.71 ± 12.7
	Total bilirubin	14	2.30 ± 0.4	13	2.38 ± 0.4	14	2.42 ± 0.4	14	2.35 ± 0.4
	CK	14	150.64 ± 119.7	13	164.77 ± 80.2	13	572.77 ± 805.1	13	249.23 ± 239.0
	Glucose	13	6.58 ± 2.0	13	7.03 ± 3.7	14	6.84 ± 3.3	14	6.39 ± 2.3
	Total protein	11	72.73 ± 6.4	9	73.11 ± 5.4	12	73.50 ± 6.4	10	72.50 ± 6.2
	Albumin	12	38.33 ± 5.5	13	40.31 ± 8.8	14	41.09 ± 8.5	12	37.58 ± 4.0
Kidney	Creatinine	14	114.14 ± 23.7	13	122.38 ± 29.8	14	114.14 ± 18.2	14	124.07 ± 32.1
	BUN	14	8.54 ± 1.9	13	8.98 ± 2.3	14	8.87 ± 1.7	14	8.50 ± 2.4
Electrolytes	Sodium	14	154.00 ± 2.6	13	152.85 ± 2.3	14	152.79 ± 2.0	14	152.43 ± 2.1
	Chloride	14	117.93 ± 3.9	13	117.38 ± 3.5	14	116.57 ± 3.7	13	117.38 ± 3.1
	Potassium	14	3.88 ± 0.3	13	3.71 ± 0.4	14	3.73 ± 0.3	14	3.79 ± 0.3
	Calcium	14	2.59 ± 0.2	13	2.59 ± 0.3	14	2.61 ± 0.3	13	2.57 ± 0.2
	Phosphorus	14	1.45 ± 0.2	13	1.37 ± 0.2	14	1.37 ± 0.1	13	1.41 ± 0.1
	Magnesium	14	0.86 ± 0.1	12	0.87 ± 0.1	14	0.86 ± 0.1	14	0.92 ± 0.2

ALP = alkaline phosphatase; ALT = alanine aminotransferase; AST = aspartate aminotransferase; BUN = blood urea nitrogen; CK = creatinine kinase

### Serum cannabinoid analysis

At week 6, the two major cannabinoids, CBD and CBDA, were consistently detected in the serum of cats receiving the CBD/CBDA-containing paste. These compounds were largely undetectable during the placebo phase, except in two cats that showed low but detectable serum concentrations of CBDA while on placebo. Of the 14 cats that completed the study, 11 had detectable concentrations of CBD and/or CBDA during the treatment phase. The median CBD concentration was 14 ng/ml (range 0–333), while CBDA concentrations were typically higher, with a median of 29 ng/ml (range 0–1088).

**Figure 5:**
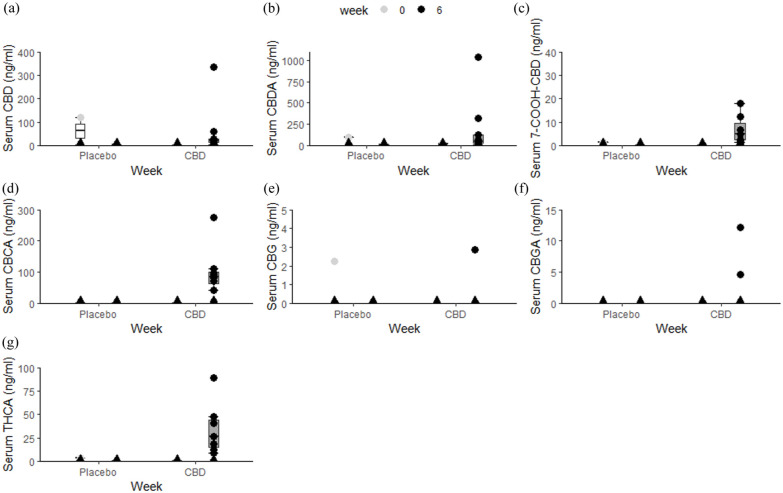
Overview of serum: (a) cannabidiol (CBD), (b) cannabidiolic acid (CBDA), (c) 7-carboxycannabidiol (7-COOH-CBD), (d) cannabichromenic acid (CBCA), (e) cannabigerol (CBG), (f) cannabigerolic acid (CBGA) and (g) tetrahydrocannabinolic acid (THCA) concentrations per treatment at baseline and week 6 of animals that completed the study (n = 14), as determined by the liquid chromatography/tandem mass spectrometry method. The box represents the median and 25th and 75th quantile. Whiskers extend from the hinge to the smallest and largest value at most 1.5 × interquartile range of the hinge. Dots represent individual observations. Triangles represent samples below detection limit

Other minor cannabinoids also increased in the treated group: CBDA rose to slightly above 100 ng/ml, cannabigerol reached 2–4 ng/ml and cannabigerolic acid peaked at 10 ng/ml. Tetrahydrocannabinolic acid concentrations remained negligible in the placebo group but reached 50 ng/ml in the treated group at week 6 (Figure 5).

## Discussion

To our knowledge, this is the first double-blind, randomised, placebo-controlled trial to evaluate the effects of a CBD/CBDA paste in cats with naturally occurring OA. The prevalence of OA in cats is high, resulting in a diminished quality of life.^10^ In a multimodal approach for treating pain and inflammation in OA, CBD is a potentially valuable treatment option. To date, research for CBD in cats with OA has been limited to only one case study.^10^

Throughout the study, DORFOP scores were lower in cats receiving CBD/CBDA paste compared with placebo, with statistically significant differences at weeks 2, 4 and 6 after starting treatment. These results suggest that the CBD/CBDA paste may effectively reduce OA pain in cats. The DORFOP questionnaire, which assesses the impact of OA pain, was used to establish an initial pain score. However, some owners did not fill in all the scores consistently; for example, because they did not have stairs at home or never brushed the cat. Since the study was a crossover design, the missing data did not impact the comparison between treatments.

The TRiP scores also showed significant improvements at week 6 in the CBD/CBDA group compared with the placebo group. These findings align with previous research where cannabinoid-rich hemp oil reduced pain reactivity in one cat with OA.^10^ The significant difference observed between the CBD/CBDA paste and the placebo after 6 weeks indicates that the CBD/CBDA paste is effective in reducing pain reactivity in cats with OA. Although joint evaluations for crepitation, effusion and thickening did not show significant changes, this may be due to the chronic and slow-progressing nature of OA. Given the study duration, it is unlikely that such changes would be detectable in a short period. Moreover, CBD is not considered a disease-modifying treatment in this context, and thus, structural changes would not be expected, despite its known anti-inflammatory properties.^14^ Likewise, the progression of OA typically takes years, and thus, significant structural changes were not expected.^5^

Detectable CBDA levels were found in most animals, suggesting sufficient dosing; however, one animal had detectable serum CBDA levels at baseline, suggesting previous CBD administration or environmental exposure to cannabinoid products. The latter has previously been reported in humans, where secondary exposure to CBD and THC resulted in detectable serum CBDA levels, potentially explaining some of the unexpected CBDA levels found in cats receiving the placebo in this study.^25^ Two animals in the placebo group at week 14 also had detectable CBDA levels, which could suggest insufficient washout between periods or external exposure. Interestingly, no differences were detected in clinical chemistry parameters between groups.

The observed variability in cannabinoid serum levels can be attributed to multiple factors. Even in pharmacokinetic studies, such as the study by Wang et al,^23^ wide interindividual variability is reported, with standard deviations spanning large ranges. In addition, serum levels are highly dependent on the timing of dosing relative to blood sampling. For example, if owners administered the dose shortly before arriving at the clinic, concentrations may be high; conversely, if a dose was missed or delayed, such as skipping the morning and evening doses before sampling, levels could fall below the limit of detection. This likely explains why three cats showed no detectable concentrations at week 6 despite being in the treatment group.^23^

Previously, Coltherd et al^16^ reported an increase in ALT after 4 weeks of CBD usage at the same overall dose as used in this study, which was also reported by Deabold et al.^18^ No significant changes in ALT levels were observed in this study in agreement with the intermittent escalation of dose findings of Kulpa et al,^17^ without elevation of the blood chemistry parameters.

Adverse events were reported, including vomiting, gagging, hypersalivation and somnolence after CBD/CBDA paste administration, which is typical for full-spectrum hemp extracts, potentially due to the strong odour and taste.^18,23,26^ However, as described above, myrcene 0.1% was added to the placebo to match the odour and taste, in order to blind the owners and researchers.

Several limitations were identified in this study. The small sample size, with a predominance of male and neutered cats, limits the generalisability of the results. In addition, the diversity of home environments could have influenced the outcomes. Future studies should aim for larger sample sizes to improve statistical power and generalisability.

The ‘caregiver placebo effect’, known to be particularly high in clinical trials involving cats, may have influenced the DORFOP scores.^27^ It is important to consider that the reaction of the cats to one of the pastes containing CBD/CBDA might have influenced the owners’ perceptions. If the cats responded more positively or more negatively to one of the pastes, whether through increased interest or other behavioural cues, there is a possibility that might have influenced the owners’ perceptions, since the subjective scoring may have been affected by recent positive or negative experiences with one of the treatments.

Many cats were reluctant to consume the CBD/CBDA paste due to the palatability. Previous studies also noted similar challenges with CBD palatability in cats.^18^ Alternative administration methods should be explored in future studies to improve compliance and reduce stress during treatment.

Inconsistent dosing was another limitation. Some owners reported difficulty in delivering the correct amount of paste using the Dial-a-Dose syringe, leading to variations in the administered dose. This may have contributed to the variability in serum cannabinoid concentrations. The wide range of CBD/CBDA levels in the cats’ blood suggests that dosing inconsistencies or missed doses may have influenced the results. Pharmacokinetic studies have shown significant interindividual variability in cannabinoid absorption and metabolism, which could account for the observed differences in serum concentrations.^23,26,28–30^

Finally, there is a lack of validated objective methods to monitor pain in cats. Two available methods, kinematic gait analysis using force plates and movement trackers, proved to be impractical in our study population. Many of the cats were unwilling to walk or showed abnormal gait patterns in the consultation room, such as crouching with their hind limbs close to the ground, which made accurate gait assessment difficult. This behaviour was likely stress-induced, as owners did not experience this gait pattern at home. Future research should explore pain assessment tools that are more adaptable to less active or stressed cats.^31^

## Conclusions

This study suggests that CBD/CBDA paste is effective in reducing pain and improving functional scores in cats with OA, as shown by significant changes in DORFOP and TRiP scores. Despite some adverse effects, the treatment showed a positive impact on pain reduction and overall function. However, the study was limited by a small sample size, inconsistent dosing and challenges with administration. Further research with larger sample sizes, longer treatment durations and alternative delivery methods is needed to confirm these findings and better understand the long-term effects of CBD/CBDA paste in cats.

## Supplemental Material

File 1 Translational Research in Pain score.

File 2 Owner-reported mobility and behaviour questionnaire.
