# Supplementary material for: Field safety and efficacy study with a cannabidiol/cannabidiol acid-rich hemp paste in cats with osteoarthritic pain
Source: J Feline Med Surg. 2025 Oct 15;27(10):1098612X251367629. doi: 10.1177/1098612X251367629 (PMC12536179; doi:10.1177/1098612X251367629)
Supplement: File 1 [file sj-docx-1-jfm-10.1177_1098612X251367629.docx]

**Supplementary file 1 – Translational Research in Pain score**

Joint and spine scoring: Scores for pain (0-4), crepitus, effusion and thickening (0-2) were recorded for all major limb joints and spinal regions using standardised palpation-based assessments.

| **R Forelimb** | **Pain (0–4)** | | **Crepitus (0–2)** | **Effusion (0–2)** | **Thickening (0–2)** |
| --- | --- | --- | --- | --- | --- |
| Feet |  | |  |  |  |
| Carpus |  | |  |  |  |
| Elbow |  | |  |  |  |
| Shoulder |  | |  |  |  |
| **R Hindlimb** | **Pain (0–4)** | | **Crepitus (0–2)** | **Effusion (0–2)** | **Thickening (0-2)** |
| Feet |  | |  |  |  |
| Hock |  | |  |  |  |
| Stifle |  | |  |  |  |
| Hip |  | |  |  |  |
| **L Forelimb** | **Pain (0-4)** | | **Crepitus (0-2)** | **Effusion (0-2)** | **Thickening (0-2)** |
| Feet |  | |  |  |  |
| Carpus |  | |  |  |  |
| Elbow |  | |  |  |  |
| Shoulder |  | |  |  |  |
| **L Hindlimb** | **Pain (0-4)** | | **Crepitus (0-2)** | **Effusion (0-2)** | **Thickening (0-2)** |
| Feet |  | |  |  |  |
| Hock |  | |  |  |  |
| Stifle |  | |  |  |  |
| Hip |  | |  |  |  |
| **Spinal Column** | **Pain (0-4)** |  |  |  |  |
| Cervical |  |  |  |  |  |
| Thoracic |  |  |  |  |  |
| T-L |  |  |  |  |  |
| Lumbar |  |  |  |  |  |
| L-S |  |  |  |  |  |

Total pain score:

Total crepitus score:

Total effusion score:

Total thickening score:

***Key***

Pain scale based on palpation (used in [1] and subsequent clinical studies)

0 **No resentment**; normal amount of movement or wriggling

1 **Mild withdrawal**; mildly resists

2 **Moderate withdrawal**; body tenses; **may orient** to site; **may vocalize** / increase in vocalization

3 **Orients to site**; **forcible withdrawal** from manipulation; may vocalize or hiss or bite

4 **Tries to escape / prevent manipulation**; **bite/hiss**; marked guarding of area

Crepitus, Effusion, Thickening: none (0); slight-moderate (1); significant-severe (2) (used in [1] and subsequent clinical studies)


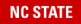
©2019 North Carolina State University, Translational Research in Pain (TRiP)
