# Supplementary material for: Field safety and efficacy study with a cannabidiol/cannabidiol acid-rich hemp paste in cats with osteoarthritic pain
Source: J Feline Med Surg. 2025 Oct 15;27(10):1098612X251367629. doi: 10.1177/1098612X251367629 (PMC12536179; doi:10.1177/1098612X251367629)
Supplement: File 2 [file sj-docx-2-jfm-10.1177_1098612X251367629.docx]

**Supplementary file 2** **– Owner-Reported Mobility and Behaviour Questionnaire**Owners rated their cats’ mobility and behaviour compared to earlier life stages using standardised questions on walking, jumping, stair use, play and grooming, with five-point response scales. Follow-up questionnaires compared changes relative to the start of the study.

| Item | Question in English | Response options |
| --- | --- | --- |
| 1 | Does your cat walk with a smooth gait, compared to when he was younger? | - Normal, as before - Not quite normal - Clearly somewhat worse than before - Barely or with great effort - Not at all |
| 2 | How does your cat jump on furniture, compared to when he was younger? | - Normal, as before - Not quite normal - Clearly somewhat worse than before - Barely or with great effort - Not at all |
| 3 | How does your cat jump off furniture, compared to when he was younger? | - Normal, as before - Not quite normal - Clearly somewhat worse than before - Barely or with great effort - Not at all |
| 4 | How does your cat walk down the stairs, compared to when he was younger? | - Normal, as before - Not quite normal - Clearly somewhat worse than before - Barely or with great effort - Not at all |
| 5 | How is the behaviour of your cat during play with pets or toys, compared to when he was younger? | - Normal, as before - Not quite normal - Clearly somewhat worse than before - Barely or with great effort - Not at all |
| 6 | Does your cat allow to be brushed, compared to when he was younger? | - Normal, as before - Not quite normal - Clearly somewhat worse than before - Barely or with great effort - Not at all |

For follow-up questionnaires, questions were changed to compare the cat to before the study and response options for every question were changed to:

- Much more often/much better compared to before the study
- More often/better compared to before the study
- The same compared to before the study
- Less often/worse compared to before the study
- Much less often/much worse compared to before the study
